# Hospital Construction

**Published:** 1897-03-13

**Authors:** 


### HOSPITAL CONSTRUCTION.

### ORMSKIRK COTTAGE HOSPITAL.

In the published plan of this hospital a door was represented too small, and as such was commented upon by our architectural referee; the architect, Mr. Beeston, informs us that the design as drawn by him shows the doorway in its proper proportions.

### CITY OF LONDON HOSPITAL FOR DISEASES OF THE CHEST.

We desire to state that the alterations and improvements favourably commented upon and described in our issue of February 27th were carried out from the designs of Mr. R. Langton Cole.

